# Pharmacy students' empathy and its determinants: a systematic review

**DOI:** 10.12688/f1000research.127017.1

**Published:** 2023-01-06

**Authors:** Hening Pratiwi, Susi Ari Kristina, Anna Wahyuni Widayanti, Yayi Suryo Prabandari

**Affiliations:** 1 Doctoral Program in Pharmacy, Faculty of Pharmacy, Universitas Gadjah Mada, Yogyakarta, Indonesia; 2 Department of Pharmacy, Faculty of Health Sciences, Jenderal Soedirman University, Purwokerto, Indonesia; 3 Department of Pharmaceutics, Faculty of Pharmacy, Universitas Gadjah Mada, Yogyakarta, Indonesia; 4 Department of Health Behaviour, Environment, and Social Medicine, Faculty of Medicine, Public Health, and Nursing, Universitas Gadjah Mada, Yogyakarta, Indonesia

**Keywords:** empathy, pharmacy students, factors, determinants, educational intervention, experience

## Abstract

**Background**: Empathy in the context of healthcare is an immersion experience to comprehend patients' viewpoints, feelings, and emotions, without passing judgment, to ensure they receive the necessary treatment to feel comfortable. Empathy for others must be possessed by healthcare professionals and healthcare students as healthcare professionals’ candidates, including the pharmacy student. This study aimed to identify and assess the determinants related to pharmacy students' empathy.

**Methods:** Three electronic databases were used for the first searches. We used peer-reviewed original papers, full text, must assess determinants that are associated with pharmacy students' empathy, and only be focused on pharmacy students (first to the fourth year) as healthcare professionals candidates. We utilized Joanna Briggs Institute Critical Appraisal Checklists to observe the quality of published publications and reduce bias.

**Results:** This review examined 14 papers that reported on determinants connected to pharmacy students' empathy. Nine studies evaluated the association between gender or sex and the level of empathy, seven studies reported educational intervention, four studies discussed the year of study, two studies explained the type of school, four studies evaluated experience, and others determinants that discussed in the included studies were career preference, intercultural sensitivity, stigma, altruism, grit, self-awareness, marital status, and family income

**Conclusions:** Educational intervention, experience, gender or sex, type of school, year of study, intercultural sensitivity, career preference, altruism, grit, self-awareness, marital status, and family income, can all have a positive impact on increased empathy among pharmacy students. We acknowledge that the included studies are heterogeneous, indicating that additional studies are necessary before reaching any firm conclusions. More research is needed to properly understand how empathy can be improved with the most effective pharmacy educational strategies. Higher levels of evidence are also required in studies to address the potential bias caused using self-report questionnaires, as well as other potential biases and inaccuracies.

## Background

Empathy, in the context of healthcare, is an emotional response experienced when attempting to comprehend patients' viewpoints, feelings, and emotions without passing judgment, to ensure they receive the necessary treatment to feel comfortable. ^1^^–^^3^ These statements demonstrate three skills of healthcare professionals: expressing empathy, cognitive ability to recognize and comprehend the views and emotions of a patient, and behavioral ability to convey this understanding. ^4^ Higher levels of empathy among healthcare professionals not only foster higher quality communication with patients, but they also result in favorable patient outcomes such as better patient self-care, higher patient satisfaction and faster recovery times. ^5^ Indeed, having empathy is crucial for building closer relationships with patients and for understanding their needs, as is frequently stated in health and educational programs for healthcare professionals. ^6^

Empathy for others must be possessed by healthcare professionals and healthcare students, as healthcare professional candidates in training, but several studies have explained that there can be a decrease in student empathy over the course of their medical training. ^7^^–^^9^ Studies indicate that empathy declines as the student progresses through medical school, ^7^^,^^8^ dentistry school, ^9^^,^^10^ and pharmacy school, ^9^^,^^11^ despite the significance of increasing empathy in health professions students. As personal discomfort from burnout, sadness, and diminished quality of life develops among trainees during their training, they are less likely to feel or show empathy. Neumann *et al.* found that deficits in the formal ( *e.g.*, lack of formal empathy training), informal (inadequate mentors and improper learning environments), and factors outside of the medical curriculum ( *e.g.*, abuse of students and high workload) medical curriculum may contribute to this decrease in empathy levels over time. ^7^ These results serve as a warning to the faculty members and educators of educational institutions that strategies must be developed not only to stop the depletion of empathy but also to improve students' empathetic orientation to better comprehend patients. ^12^ To develop a strategy, one must define the target needs and understand the factors or determinants that are related to student empathy, in this case being the pharmacy student. This review is designed to identify those of the pharmacy student. The growth of the pharmacy educational system was anticipated to broaden pharmacists' roles in patient-centered care. ^2^^,^^13^ Because empathy is important for patient care, educational strategies should encourage the development of empathy during training.

A previous systematic review was conducted by Maximiano-Barreto *et al.* to investigate the variables linked to empathy levels among health-related students and professionals. ^6^ However, in the included studies there was not much evidence to indicate pharmacy students' empathy levels specifically. Quince *et al.* conducted a review in a similar context to determine the variables that could influence how empathy develops in medical students, ^14^ although the scope of this review was limited by its predominant emphasis on medical students, whereas in this review the emphasis is placed more on the empathy of pharmacy students. The authors were interested in conducting a comprehensive review of determinants related to empathy with a focus on pharmacy students to fill this gap in the literature.

This study aimed to identify and assess the determinants related specifically to pharmacy students' empathy. This review sought to answer the following two questions:

1) How is empathy measured?
2) What determinants may relate to the development of pharmacy students’ empathy?

## Methods

In the early planning stage, the four authors (HP, SAK, AWW, and YSP) discussed the existing theory regarding the determinants related to students' empathy. The author reads several articles that discuss factors that may be related to student empathy. Previous studies identified “perspective-taking,” “compassionate care,” and “standing in patient's shoes” programs that provide healthcare students with information about the relevance of empathy and its role in patients treatment. ^15^^,^^16^ Organizational culture, personal and interpersonal relationships, and demographic characteristics are all highly linked to increased empathy. The authors were interested in conducting a comprehensive review of determinants related to empathy with a focus on pharmacy students. ^16^ This review followed the Preferred Reporting Items for Systematic Reviews and Meta-Analyses (PRISMA) guidelines. ^17^

### Eligibility criteria

We used the following inclusion criteria to evaluate each of the articles under consideration to respond to our research questions:

1) They must be peer-reviewed original papers.
2) They must be published in full text.
3) They must only be focused on pharmacy students (first to the fourth year) as healthcare professionals candidates.
4) They must assess determinants that are associated with pharmacy students' empathy.
5) The subdivisions of empathy must include attitude, behavior, and perception.
6) They must conduct design research, such as cross-sectional studies, cohort studies, case-control, randomized control trials, or quasi-experimental studies.

Excluded from the systematic review were studies on animals, review articles, qualitative studies, commentary articles, letters to the editor, conference abstracts, books, guidelines, and theses.

### Information sources and search strategy

The initial searches were conducted using three electronic databases: PubMed, Science Direct, and Scopus. From July 14, 2022, to the latest recent search on July 16, 2022, we considered articles on factors or determinants about pharmacy students' empathy from this three-database collection. There were no restrictions on publication ages throughout this period. Each database used the terms “factors” AND “pharmacy students” AND “empathy” as a single search term or in combination using Medical Subjects Heading (MeSH) terms with the Boolean operators. We also searched for additional reference materials by consulting the cross-references listed in the included publications via Google Scholar on August 16, 2022.

### Study selection and data extraction

**Figure 1.**
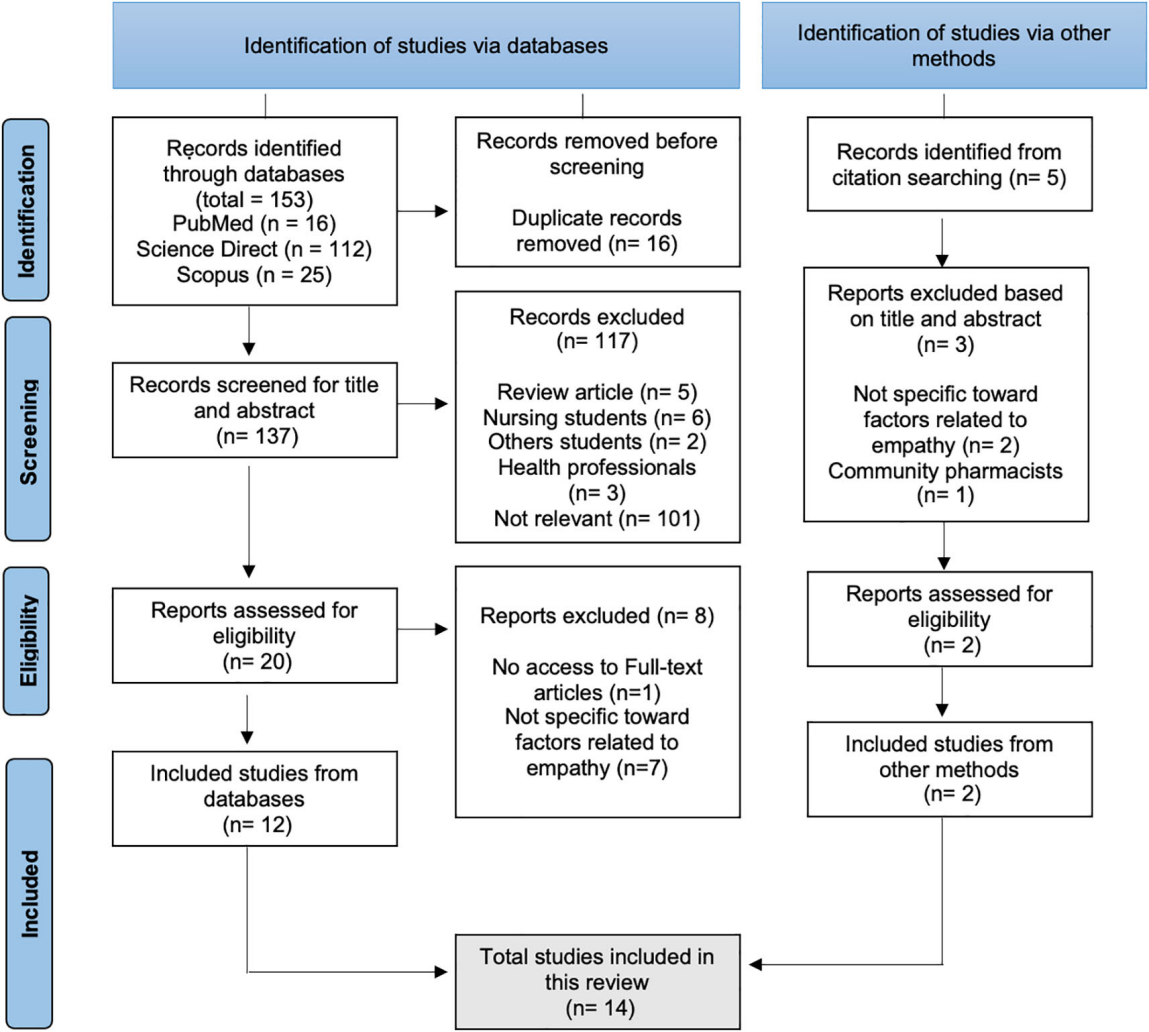
PRISMA diagram of this review.

The retrieved studies from databases were screened for their title and abstracts by three authors (HP, SAK, and AWW) to ensure they fit the eligibility criteria. We used a common data extraction spreadsheet (Microsoft Excel) to chart the necessary data. To eliminate duplication, Mendeley Reference Manager was used to obtain every article. Next, full texts of selected studies were reviewed by three authors (HP, SAK, YSP) to determine their relevance. Discrepancies whether the title, abstract, and entire manuscript meet the inclusion and exclusion criteria were resolved through discussions between authors until concordance was obtained. A PRISMA diagram was used to record the screening and selection process ( Figure 1).

A data extraction was conducted by the two independent reviewers (HP and SAK) to obtain the key study information. Included studies should provide a primary focus on identifying the determinants which related to pharmacy student empathy. While other supporting variables include characteristics of included studies (the year of publication, country, study design, participants, and type of participants) and measurements adjusted for eligibility criteria.

We created a spreadsheet with details about the publication year, country, study design, participants, participant types, measurements, and factors related to empathy. Two authors (HP and SAK) read the entire paper and highlight the key points discussed inside it, with adjustments for the relevant variables. Furthermore, this pertinent information was reviewed in the results section. The two reviewers' disagreement (HP and SAK) were settled through discussion with another member of the research team (HP, SAK, AWW, YSP) and HP as the person in charge of this discussion process.

One point of disagreement is the assumption that the “country” is related to the country in which the study was conducted rather than the country of the affiliated university. Another example is figuring out the essential factors that related pharmacy students' empathy as indicated in the included studies.

### Quality assessment

We utilized Joanna Briggs Institute Critical Appraisal Checklists (JBI) according to the methodological design of the studies: cross-sectional studies, cohort studies, case-control, randomized control trials, and quasi-experimental studies. This is performed to observe the quality of published articles. The JBI critical appraisal method was used to determine the extent to which the included studies attempted to reduce risk of biases related to the appropriateness of the study objectives participant selection, data collection, data analysis, randomization, treatment allocation, blinding, and interpretation of results. There were 8 questions for a cross-sectional study design, 11 questions for a cohort study, 10 questions for a case-control study, 9 questions for a quasi-experimental study, and 13 questions for a randomized controlled trial.

Appraisal of studies was undertaken by two independent authors (HP and SAK) and assessments were compared. The discrepancies were discussed with other research team members (AWW and YSP). Examples of discrepancies include checking to see if the inclusion criteria were explained clearly and checking to see which confounding variables were mentioned in the article.

Each checklist criterion was rated as yes”, “no”, “unclear”, or “not applicable”. When the studies reached up to 49% of the “yes” score, the risk of bias was classified as high; moderate when they reached 50 to 69% of the “yes” score; and low when they reached more than 70% of the “yes” score (Joanna Briggs Institute Critical Appraisal Checklists).

## Results

Initially, a total of 153 records were retrieved from databases and other methods (PubMed with 16 articles, Science Direct with 112, Scopus with 25, and citation searching from Google Scholar with 5). Titles and abstracts were evaluated after duplicate articles (137 articles from databases) were removed to see how they related to this systematic review. The study titles and abstracts were reviewed according to inclusion criteria *i.e.,* they must be focused on pharmacy students (first to the fourth year) as healthcare professionals’ candidates, and they must assess determinants that are associated with pharmacy students' empathy. In cases where there was uncertainty regarding whether the title and abstract adequately express the determinants related to pharmacy students' empathy, the full text was read.

Disagreements among the reviewers (HP, SAK, and AWW) on whether the title and abstract met the inclusion requirements were settled through discussion until consensus was reached. We held discussions by getting together and going over titles and abstracts that would meet our inclusion criteria. We read and discuss the entire article together, considering the key points for entering the included studies until we get to an agreement if an article is identified that is doubtful considering the inclusion criteria. Out of these, 120 items were excluded at this stage because they failed to fulfill the requirements for inclusion, including review articles, publications with populations of non-pharmacy students, and articles that were not pertinent to our topic. For example, several studies focus more on nursing students, ^18^^–^^20^ another study focuses more on dietetic students. ^21^

A total of 22 articles were given a complete text examination and eligibility evaluation *i.e.* they must be peer-reviewed original papers, they must be published in full text, they must be focused on pharmacy students (first to the fourth year) as healthcare professionals’ candidates, and they must assess determinants that are associated with pharmacy students' empathy. The research team members (HP, SAK and YSP) independently reviewed the full texts of articles that fulfilled inclusion criteria and passed the title and abstract evaluation. The source population, participants type, sample size, study design, outcome measurement type, and determinants related to empathy were just a few of the metrics that were evaluated for each article. The research team's discussion led to the extraction and finalization of the data. We read and discussed the full text's information, particularly whether it included information on factors that influence empathy as well as supplementary details like the included studies' characteristics (year of publication, country, study design, participants, and type of participants) and measurements. Finally, 14 articles were included in this systematic review. Figure 1 provides an overview of the selection process.

Each study that was considered, including an RCT, a quasi-experiment, and cross-sectional studies, had its quality and risk of bias evaluated critically. All studies obtained more than 50% “yes” responses on the checklist, and the risk of bias was graded as low, according to the author's quality evaluation using the JBI Quality appraisal checklist. The results of the quality assessment are shown in supporting information in data availability section. ^22^

### Characteristics of included studies

The included studies were published between 2011 and 2022 and were conducted in seven countries: eight in the United States ^2^^,^^23^^–^^29^ and one each from Korea, ^30^ China, ^31^ United Kingdom, ^32^ Indonesia, ^33^ Singapore, ^34^ and Iran. ^35^ Eleven of the included studies used cross-sectional designs, two studies used randomized control trial designs, ^24^^,^^34^ and one of the studies used one group pretest-posttest intervention design. ^28^

Twelve (86%) studies included pharmacy students as participants and two (14%) studies informed pharmacy students and other health professional students or pharmacists. ^28^^,^^33^ Pharmacy students as participants ranged from first year through to fourth year students. Three of the included studies reported first-year level of pharmacy students as participants. ^2^^,^^26^^,^^29^ Three studies reported second-year pharmacy students as participants. ^23^^,^^24^^,^^34^ Two studies informed first through to fourth year students as participants. ^31^^,^^32^ We found one paper focussed each on the third year, ^25^ the fourth year, ^33^ the second through to third year, ^30^ the first through to third year, ^27^ and two others that did not specify the education level of students were included in the study. ^28^^,^^35^ The number of participants included in each of the studies ranged from 40 to 1013 pharmacy students.

### Outcome measures

All studies evaluated participant improvements in empathy using self-report measures. Self-report measurements typically consisted of a single question or a self-report survey. There were several different self-report survey formats used in included studies. The most frequently used instrument was the Jefferson Scale of Empathy for Health Professions Students (JSE-HPS). ^2^^,^^24^^,^^28^^–^^32^^,^^34^^,^^35^ The JSE-HPS instrument contained 20 items with response options based on a 7-point Likert scale and measuring Perspective Taking, Compassionate Care, and Standing in the Patient's Shoes. Another instrument widely used in included studies was the Kiersma-Chen Empathy Scale (KCES). ^26^^,^^28^^,^^33^ The KCES, a 15-item survey with a 7-point Likert-type scale, measured two aspects of empathy: the capacity to comprehend others' perspectives (cognitive domain) and the capacity to empathize with their thoughts and experiences (affective domain). Table 1 provides specifics about the empathy measurement tools used in included studies.

**Table 1.** Summary of characteristics of included studies.

No	Authors	Year	Country	Study design	Participants	Participants type	Measures	Factors that related with empathy
1	Fjortoft *et al.*	2011	United States	Cross-sectional	187 pharmacy students	First-year students of Chicago College of Pharmacy at Midwestern University	The Jefferson Scale of Empathy-Health Profession Students version (JSE-HPS)	**Gender** Female pharmacy students scored substantially higher on average empathy than male colleagues.
2	Sales *et al.*	2013	United States	Quasi-experimental	84 pharmacy students (26 respondents in the lecture group, 30 in the case-scenario group, and 28 in the simulation group)	Second-year pharmacy students at the University of Pittsburgh School of Pharmacy	The cultural assessment survey instrument	**Educational intervention** Respondents in the simulation group were more likely to agree or strongly agree with the item of the cultural desires on the post-intervention survey instrument, indicating that they would like to learn about diverse cultures and ethnic groups' health beliefs and practices. In the case-scenario group, there was a considerable change in the cultural awareness component related to mastery of cultural competency. In the lecture group, there were significant changes in the cultural skills question regarding modifying one's communication style, demeanor, and interviewing questions during cultural encounters and in the cultural empathy question emphasizing the importance of pharmacists addressing medication issues from the patient’s personal and cultural perspective when providing care.
3	Lor *et al.*	2015	United States	RCT	40 pharmacy students (intervention group (20) and control group (20))	Second-year student pharmacists	The JSE-HPS	**Educational intervention** These studies found an increase in empathy levels right after the intervention. A considerable increase in empathy levels after 7 days, but no significant differences after 90 days.
4	Jeon and Cho	2015	Korea	Cross-sectional	447 pharmacy students	Second and third years pharmacy students at five Korean universities	The Korean-translated JSE-HPS version and the IRI	**Gender, year of study, future career preference** There were no significant differences based on gender, year of study, or predicted career preference. **School-wide characteristics** Except for the standing in the patient's shoes subscale, there were significant differences across all measures based on whether students were from women's or co-ed universities. Students in women's universities reported better levels of perspective taking, compassionate care, and general empathy than students at co-ed universities. Pharmacy students at private institutions indicated greater levels of overall empathy than students at public universities.
5	Li *et al.*	2015	China	Cross-sectional	263 pharmacy students	First to fourth year pharmacy students at Wuhan University of Science and Technology	The JSE-HPS	**Year of study** The fourth-year students' mean empathy score was much higher than any of the prior years, whereas second-year students had the lowest empathy ratings. **Gender, age, and career preference** Gender, age, and career preference differences were not statistically significant. **Educational intervention** The participants' preferred humanistic education implementation option among pharmacy undergraduate students It was widely assumed that strengthening and integrating humanities into professional education contributed to an improvement in empathy, professionalism, and self-care.
6	Hall *et al.*	2015	United Kingdom	Cross-sectional	318 pharmacy students	All undergraduate pharmacy students at Queen’s University Belfast	The JSE-HPS version	**Gender** Females had a slightly higher mean empathy score than males, but the difference was not statistically significant. **Year of study** Respondents at higher levels had considerably higher mean empathy scores. Level 4 had the greatest score, while level 1 had the lowest. **Part time employment status** The mean empathy score for students with part-time jobs was slightly higher than for those without, but there was no statistically significant difference between the two groups. **Health status** There was no statistically significant difference in mean empathy scores between those with chronic medical conditions and those who did not.
7	Kerr *et al.*	2015	United States	Quasi-experimental	48 pharmacy students	The third professional year in a standard four-year professional degree after successfully completing the required endocrine and cardiovascular pharmacotherapeutic courses	The Jefferson Scale of Empathy (JSE)	**Educational intervention (simulation)** Over the 6-week encounter, total JSE scores climbed dramatically from an average of 114 to 123.
8	Ekong *et al.*	2017	United States	Cross-sectional	134 pharmacy students	First-year PharmD students enrolled in a 4-year doctor of pharmacy program at one dual-campus pharmacy school	The KCES and the ISS	**Sex** The independent samples t-test revealed a significant relationship between empathy and sex, with females reporting higher levels of empathy than males. **Intercultural sensitivity** The Pearson correlation demonstrated a strong relationship between tendency for empathy and intercultural sensitivity, with higher attitudes toward empathy associated with a higher predisposition for intercultural sensitivity.
9	Williams *et al.*	2020	United States	Cross-sectional	318 pharmacy students	First- through third-year Doctor of Pharmacy students	The JSE	**Curriculum year** There were no significant differences in median JSE scores between student groups based on curriculum year. **Gender, direct patient care experience, chronic illness caregiver experience** There were no significant differences between male and female students, students with and without direct patient care experience, or students with and without chronic illness caregiver experience.
10	Simko *et al.*	2021	United States	Quasi-experimental	178 students (50% Bachelor of Science in Nursing; 23% Pharmacy Doctorate students; and 27% a mix of healthcare majors	Pharmacy, nursing students, and mix healthcare major	The JSE-HPS and the KCES	**Educational intervention** The observed gains in empathy scores suggest that the CPSETKit was effective in enhancing empathy among health care students for patients suffering from chronic pain.
11	Fong *et al.*	2021	Singapore	RCT	130 pharmacy students (46 participants in the didactic lecture group, 40 participants in the jigsaw group, and 44 participants in the fishbowl group)	Second-year pharmacy students at National University of Singapore pharmacy undergraduates	The JSE-HPS version	**Educational intervention with different debrief method** The JSE-HPS scores improved significantly after the workshop, both within each debrief and overall. However, males in the fishbowl group increased much more than females after the session. There was also a significant difference at baseline between participants in voluntary programs and those who were not in the jigsaw group. **Gender** Males scored substantially lower than females at baseline in “compassionate care,” but this difference was not significant in pre/post-workshop score changes.
12	Sianturi *et al.*	2021	Indonesia	Cross-sectional	1013 pharmacy students and 250 pharmacist	All the final-year pharmacy students who were on hospital training (nine universities in Indonesia)	Adapted questionnaire consisting of 15 items, The Kiersma-Chen questionnaire	**Stigma and interaction with patients** In addition to being strongly related with inadequate knowledge and less empathy, pharmacists who met with patients rather than students were significantly associated with stigma.
13	Reed *et al.*	2021	United States	Cross-sectional	208 pharmacy students	First-year Doctor of Pharmacy students enrolled in professionalism courses at the University of Maryland School of Pharmacy or the University of Mississippi School of Pharmacy during the 2018-2019 academic year	The JSE-HPS	**Educational intervention** This study found no substantial long-term gain in empathy among student pharmacists after completing longitudinal professionalism courses. **Altruism, grit, self-awareness** The relationship between empathy and altruism was modest to moderate. Empathy was also associated with grit but not with self-awareness or locus of control. **Age, gender, prior health care experience** Age, female gender, and prior health care experience were all significant predictors of baseline empathy in the total sample. The overall model fit was excellent.
14	Fashami et al.	2022	Iran	Cross-sectional	504 pharmacy students	Pharmacy students in five pharmacy schools in Iran	The Farsi-translated version of the JSE-HPS	**Gender** Females scored substantially higher on total empathy than males. Females also shown much greater empathy for compassionate care and perspective taking. **Marital status, year of study, type of school, family income** There were no statistically significant changes in empathy scores and its aspects based on marital status, year in pharmacy program, type of school, or family income.

RCT: Randomized Control Trial, The JSE-HPS: The Jefferson Scale of Empathy-Health Profession Students version, The IRI: the Interpersonal Reactivity Index, The KCES: The Kiersma-Chen Empathy Scale, The ISS: Intercultural Sensitivity Scale.

### Determinants related to the pharmacy students’ empathy

*Gender or sex*

Among the included studies in this review, eight studies evaluated the association between gender and the level of empathy ^2^^,^^27^^,^^29^^–^^32^^,^^34^^,^^35^ and one study evaluated the association between sex and the level of empathy. ^26^ Most of them concluded that the overall empathy score in female students was significantly higher than that in males, ^2^^,^^26^^,^^29^^,^^32^^,^^34^^,^^35^ with two included studies discovering no discernible differences between them. ^30^^,^^31^

In 2022, Fashami *et al*. conducted a cross-sectional study to measure the empathy score among 504 pharmacy students in five Iranian pharmacy schools. ^35^ According to this study, female students scored much higher on empathy than males did overall. Additionally, in the JSE-HPS, females had considerably greater empathy scores in the compassionate care and perspective-taking domains. At the Midwestern University Chicago College of Pharmacy in 2011, Fjortoft *et al*. performed a survey of 187 first-year pharmacy students. ^2^ According to this study, female pharmacy students scored much better on average for empathy than their male colleagues did. Jeon and Cho’s findings contrasted this by stating in their research that there were no noteworthy gender differences. ^30^

*Educational intervention*

Seven included studies reported that educational intervention can become one of the factors or determinants related to the empathy of pharmacy students. ^23^^–^^25^^,^^28^^,^^29^^,^^31^^,^^34^ Pharmacy students can develop their empathy using a variety of educational interventions, including but not limited to a learning module, role-reversal exercises, patient simulations, workshops, dramatic performances, and games involving patients’ medication. ^36^ Lor *et al.* conducted a randomized study in which participants in the intervention group underwent a three-day simulation of a specific activity and received daily debriefings from faculty members. ^24^ The three exercises involved were: simulating losing one's dominant hand (participants had to wrap their dominant hand in gauze and were not permitted to use it); simulating losing one's sight (participants had to wear sleep masks); and simulating losing one's ability to speak (participants were only allowed communicate using a whiteboard and marker). Seven days after the intervention, participants who were randomly assigned to a single, three-day empathy intervention experienced a significant rise in empathy scores.

Simko *et al.* used the apparatuses contained in a Chronic Pain Simulation Empathy Training Kit (CPSETKit) to simulate chronic pain from several disease states. ^28^ The purpose of this study was to assess how using a Chronic Pain Simulation Empathy Training Kit affected the empathy of pharmacy, nursing, and health sciences students (CPSETKit). The use of the CPSETKit increased the empathy for chronic pain patients in health care students. Additionally, Reed *et al.* investigated whether first-year pharmacy students' empathy levels enhanced after taking a longitudinal professionalism course at two pharmacy schools. The outcomes demonstrate that continuous professionalism training at two pharmaceutical schools raised the level of pharmacy students' empathy. ^29^

*Year of study and type of school*

Of the 14 included studies, there were four studies ^30^^–^^32^^,^^35^ discussed the year of study that related to the empathy of students, and two studies ^30^^,^^35^ explained that the type of school related to the students’ empathy.

Li *et al*. reported that in comparison to any prior years, the fourth-year students' mean empathy score was significantly higher than in other years but did not differ much. ^31^ The second-year students also scored the worst on empathy. The fourth-year class had higher empathy scores than the first-year class, but there was no statistically significant difference in empathy scores by age, which may be because the seniors have finished all of their professional coursework and clinical practice. Due to the fact that pharmacy students in China spend their first three years away from the clinical setting, they seldom come into contact with clinical role models until their last year of training. Students work with patients during their fourth-year clinical clerkships while receiving training in ethics, practice management, and the management and treatment of fearful patients. As a result, they may come at this stage to deeply and vividly understand the significance of the relationship between pharmacists and patients.

On the other hand, Jeon & Cho reported that even though there were no differences across the students’ years of study in their research, the type of university attended determined significant differences in the total empathy score. ^30^ Depending on whether the students came from co-educational or women's universities, significant variations were found for all variables. Students in women's universities exhibited higher levels of empathy overall, compassionate care, and perspective-taking than those at co-educational universities. Since 2011, the four-year BSc pharmacy degree in Korea has been replaced by a two+four hour Doctor of Pharmacy (PharmD) program. The first graduates of the new program will receive their diplomas in 2015. Students could take the Pharmacy Education Eligibility Test (PEET) if they have completed the minimum number of pre-pharmacy courses (two years).

*Experience*

A total of four studies of included studies evaluated the association between experience and the level of empathy. ^27^^,^^29^^,^^32^^,^^33^ According to Williams *et al.*, there were no appreciable differences in the cognitive empathy of student groups with direct patient care or chronic illness caregiving experiences. ^27^

Similar to Hall *et al.*, part-time employment experience affect students' empathy scores, even though mean scores were higher for part-time employees compared to non-employees. ^32^ Additionally, students who said they worked with patients had a slightly higher mean empathy score than students who had not worked with patients. These findings are significant because they imply that students who have more patient exposure are not adversely affected by patient contact.

Reed *et al.* explained that through subgroup analysis of student employment status, *i.e.*, whether they worked or did not work throughout the school year, the latter were similarly more likely to analyze an increase in empathy with time, providing additional support for the moderating influence of health care experience. ^29^ Furthermore, Reed *et al.* confirmed that there might be chances to tailor training to students' prior experience or levels of fundamental empathy. Teaching that aims to foster empathy might focus on individuals who are most in need given the growing number of abilities that pharmacy schools are expected to cultivate in student pharmacists (although time and resources are limited).

A cross-sectional study on HIV-stigma was undertaken by Sianturi *et al.* in 2022, using pharmacists and pharmacy students as participants. The findings indicate that pharmacists were more knowledgeable about HIV therapy and shown greater empathy than pharmacy students. These discrepancies in knowledge and empathy may be brought on by the fact that many pharmacy students lack any prior patient-care experience.

*Other*

Other determinants that discussed in the included studies were career preference, ^30^^,^^31^ intercultural sensitivity, ^26^ stigma, ^33^ altruism, grit, self-awareness, ^29^ marital status, and family income. ^35^

In the context of pharmacy, empathy may also differ based on career preference. Two studies discussed career preference, and both reported that in terms of students' preferred future careers, there were no significant differences in empathy. ^30^^,^^31^ Future research should clarify whether pharmacists' empathy levels vary depending on their professional field, even though no significant mean differences were found between pharmacy students' preferred future careers in this current study.

A cross-sectional study on HIV-stigma among healthcare professionals was undertaken by Sianturi *et al.* in 2022, using both pharmacists and pharmacy students as participants. ^33^ This stigma has been characterized as the irrational belief, unfavorable behavior, and negative attitude toward patients due to their HIV status. The findings indicate that compared to students, pharmacists demonstrated greater empathy and knowledge about HIV treatment. Many pharmacy students lack patient care experience, which could account for the disparities in knowledge and empathy. Lower levels of empathy and inadequate HIV knowledge were substantially associated to stigma.

Altruism, grit, and self-awareness were additional factors that were perceived to be related to empathy scores. According to Reed *et al.* there is a weak to moderate correlation between empathy and altruism. Along with grit, empathy was tangentially associated with neither self-awareness nor locus of control. ^29^ Ekong *et al*. showed that the Pearson correlation demonstrated a strong association between higher attitudes toward empathy and a higher propensity for intercultural sensitivity. This relationship was like that with intercultural sensitivity. ^26^ On the other hand, to Fashami *et al.* found no statistically significant variations were found between empathy scores and its dimensions with regard to marital status and family wealth, in their 2021 study. ^35^

## Discussion

The objective of this systematic review was to identify and assess the determinants related to pharmacy students' empathy. This review revealed a clear trend between studies on the factors that related to the empathy of pharmacy students, despite variations in study design, data collection, and the results.

One determinant from this review that deserves attention is that females appear to exhibit a higher level of empathy than males. Some included studies stated that female students had a higher level of empathy than male students, while other studies explained that there was no discernible difference between the two groups in terms of empathy. According to Klein & Hodges, there are no aptitude differences between males and female that could account for gender disparities in empathy levels. ^37^ They contend that practically everyone may develop greater empathy if they are given the right motivation. While Bratek *et al.* evaluated the level of empathy among medical school students, trainees, residents, and specialists, the results revealed that female participants had greater empathy scores than male participants. ^38^ The existence of sex-based differences in the levels of empathy displayed by healthcare personnel indicates the potential benefits of targeted reinforcement of the honed abilities for empathetic responses during patient-centered communication skills training.

Other sociodemographic factors identified were the year of study and type of school. Our findings revealed that while some studies reported that there was no significant difference between the study years, others claimed there was a considerable increase in empathy in the fourth year compared to the prior year. These findings are comparable to those from Magalhães *et al.*, who found that sixth-year students scored more highly on empathy scores than first-year medical students. ^39^ They raise the prospect that restrictions might have been raised during medical training. These variations may be caused by different levels of clinical exposure throughout undergraduate courses, different teaching strategies, or cultural variations. ^32^ The type of school is also associated with the level of empathy among pharmacy students. According to one of the included studies, students in women's universities showed more overall empathy than those at co-educational universities. ^30^ This may be connected to the instructional environment at each school, or the social environment.

Beyond the determinants described above, educational interventions, experience, and career preference also exert an influence on pharmacy students’ empathy. The result suggest implementation of educational intervention in the institution's curriculum is crucial. According to a systematic review by Batt-Rawden *et al*., educational interventions can be successful in upholding and fostering empathy in undergraduate medical students. ^3^ Furthermore, they emphasize the requirement for multicentre, randomized controlled trials that disclose long-term data to assess the durability of intervention effects. Similarly, Pratiwi *et al.* showed that educational interventions can successfully encourage empathy in pharmacy students. The majority of the study that is included use experiential training techniques like simulations, role-playing, or learning based on conceivable scenarios or games to increase participants' empathy. ^40^ The study of health professional students’ empathy levels enables us to determine whether it can be used to predict the choice of university course and whether it is a stable quality or changes during the course curriculum. As an example, research done by Saiva *et al*. in 2020 suggests simulation may be a useful educational tool to include in health curriculum. According to the JSE score, this study discovered a considerable improvement in empathy for the elderly population following the simulation. ^41^

In the context of pharmacy, the studies reviewed argue that empathy may also differ depending on the preferences for future career characteristics. For instance, pharmacists working in community pharmacies may exhibit more empathy than those who work in hospitals because they have more opportunities to interact directly with patients. ^30^ Our review also demonstrated how the experience has a significant impact on pharmacy students' capacity for empathy. This was also covered by Tisdale *et al.*, who found that medical students' empathy levels considerably increased right away following their patient interaction experience and that this the persisted for five weeks. ^42^ This is similar to findings in Boarman *et al.*, which described involvement in geriatric experience and resulted in a statistically significant improvement in pharmacy students’ empathy scores toward geriatric. ^43^ Additionally, survey findings show that encounters with elderly patients at a single event improved students' comfort levels during screening, counseling, and communication.

This review successfully pinpoints factors or determinants that are associated with pharmacy students' empathy. However, it has some limitations. We acknowledge that the included studies are heterogeneous, indicating that additional studies are necessary before reaching any firm conclusions. Second, because most of the studies that were included measured empathy using self-reports, they did not necessarily accurately capture the empathy of pharmacy students and how patients felt. Understanding how the patient feels and the patient's situation is critical for pharmacy students to grasp the significance of empathy for patients. It is highly advised to conduct qualitative research to obtain a more accurate understanding of the patient's feelings and empathy. Higher levels of evidence are also required in studies to address the potential bias caused by the use of self-report questionnaires, as well as other potential biases and inaccuracies. Thirdly, considering articles written in various languages would have stimulated new ideas about how pharmacy students’ differences affect empathy levels. More research is needed to properly understand the ways by which empathy levels can be improved and to develop the most effective pharmacy educational strategies.

## Conclusions

This review examined 14 papers that reported on determinants connected to pharmacy students' empathy. According to the findings of this systematic review, a variety of determinants, including educational intervention, experience, gender and/or sex, type of school, year of study, intercultural sensitivity, career preference, altruism, grit, self-awareness, marital status, and family income, can all have a positive impact on increased empathy among pharmacy students. This review came up with a few suggestions for future research projects. A deeper investigation into the effects of educational interventions, experience, gender, school type, year of study, intercultural sensitivity, career preference, altruism, grit, self-awareness, marital status, and family income is recommended. The establishment of educational practices for the development of empathy in teaching and professional settings is necessary taking into account various determinants that are relevant with empathy, especially for pharmacy students who will be in direct contact with their patients.

## Data Availability

All data underlying the results are available as part of the article and no additional source data are required. Repository: PRISMA flow chart for “Pharmacy students’ empathy and its determinants: a systematic review. https://doi.org/10.6084/m9.figshare.21259527.v1. Repository: PRISMA checklist for “Pharmacy students’ empathy and its determinants: a systematic review. https://figshare.com/articles/dataset/Untitled_Item/21458382?file=38528501. Repository: Joanna Briggs Institute Critical Appraisal Checklists according to the methodological design of the studies. https://figshare.com/articles/dataset/Critical_Appraisal/21455916. Data are available under the terms of the Creative Commons Attribution 4.0 International license (CC-BY 4.0).
