# Supplementary material for: Pharmacy students' empathy and its determinants: a systematic review
Source: F1000Res. 2023 Jan 6;12:18. [Version 1] doi: 10.12688/f1000research.127017.1 (PMC11399769; doi:10.12688/f1000research.127017.1)
Supplement: Peer review history

# Reviewer report: Reviewer response for version 1

**DOI:** 10.5256/f1000research.139482.r159584

**Published:** 2024-09-13

**Authors:** Tuangrat Podha

**Affiliations:** 1 Faculty of Pharmacy, Thammsat University, Bangkok, Thailand

This manuscript is quite complete in providing the detail of a comprehensive review which allow replication by others.

 The finding from systematic review of Pharmacy students' empathy and its determinants could be generalized to other contexts.

 The finding of this study could be generalize to other context so that the other researchers who are interested in measuring and developing empathy in health science studies can learn from this study. The conclusion of the results is clear and useful for the development of empathy in teaching of the pharmacy students those will be an important part of healthcare team.

Are the rationale for, and objectives of, the Systematic Review clearly stated?

Yes

Is the statistical analysis and its interpretation appropriate?

Not applicable

Are sufficient details of the methods and analysis provided to allow replication by others?

Yes

Are the conclusions drawn adequately supported by the results presented in the review?

Yes

Reviewer Expertise:

Health economic, Health service research, Health policy

I confirm that I have read this submission and believe that I have an appropriate level of expertise to confirm that it is of an acceptable scientific standard.

# Reviewer report: Reviewer response for version 1

**DOI:** 10.5256/f1000research.139482.r199443

**Published:** 2023-08-31

**Authors:** Fatemeh Mirzayeh Fashami

**Affiliations:** 1 McMaster University, Hamilton, Ontario, Canada

Thank you for giving me the opportunity to review this manuscript. 

 Background:

 1. You did not answer the question, “How is empathy measured?” You answered, “How is empathy measured in pharmacy students” Please add that.

 2. I think it would be valuable to add data regarding the most common tools to measure empathy in pharmacy students in the introduction.

 Method:

 3. This is your statement:

 The retrieved studies from databases were screened for their title and abstracts by three authors (HP, SAK, and AWW) to ensure they fit the eligibility criteria.

 It is not clear if the reviewers reviewed each article independently or not. Please indicate.

 According to the PRISMA checklist:

 Specify the methods used to collect data from reports, including how many reviewers collected data from each report, whether they worked independently, any processes for obtaining or confirming data from study investigators, and, if applicable, details of automation tools used in the process.

 4. Your last search was one year ago. You need to update your search and include the studies that were published from 2022 to 2023.

 5. What is your justification for only including 1 ^st^  to 4th-year students? Please indicate that. Based in different countries, there are bachelor's and master of pharmacy or pharm.D degrees. In some countries, both master's and pharm.D degrees exist, but in some others, only one of them is applicable. By excluding by years in pharmacy programs,  what will happen to the countries that have 6-year pharm.D programs? (Like Iran) or what will happen to the students who are in 4-year programs but have delays in graduation and are now in 5 ^th^ year?

 6. Generally, you need to talk about the questionnaires and how empathy is measured. JSE is a standardized tool for measuring empathy, and you need to explain that.

 Results:

 7. PRISMA diagram:

 You should describe the PRISMA diagram according to your defined PICO. Instead of reviewing articles and indicating design, you can combine all the non-pharmacy students in one category. Also, you should indicate why 101 hits were not relevant (due to population, design, or outcome…)

 8. Related paragraph:

 The study titles and abstracts were reviewed according to inclusion criteria i.e., they must be focused on pharmacy students (first to the fourth year) as healthcare professionals’ candidates, and they must assess determinants that are associated with pharmacy students’ empathy. In cases where there was uncertainty regarding whether the title and abstract adequately expressed the determinants related to pharmacy students’ empathy, the full text was read.

 Disagreements among the reviewers (HP, SAK, and AWW) on whether the title and abstract met the inclusion requirements were settled through discussion until consensus was reached. We held discussions by getting together and going over titles and abstracts that would meet our inclusion criteria. We read and discussed the entire article together, considering the key points for entering the included studies until we get to an agreement if an article is identified that is doubtful considering the inclusion criteria.

 This part is not relevant to the results. Move it to method. Also, there are repetitive parts in results that are focused on the method section. You have to move all of them to method and ONLY talk about results. The PRISMA diagram is presented to prevent explaining too much in the results section.

 9. Reword this section:

 Out of these, 120 items were excluded at this stage because they failed to fulfill the requirements for inclusion, including review articles, publications with populations of non-pharmacy students, and articles that were not pertinent to our topic. For example, several studies focus more on nursing students,18–20 another study focuses more on dietetic students.21

 Out of X number of hits from databases and grey literature search, Y were excluded mainly due to duplicate (n1), study design (n2), different population (n3) …

 10. In Table 1, you indicated that Fashami et al reported no significant changes in empathy score and pharmacy year. In the test, you have mentioned that this study did not include education level. This reference included the educational level of students, and you can see that in “Table 1. Demographic Characteristics of the Study Population”. Please revise this section and all the related parts.

 11. In this paragraph, A total of four studies of included studies evaluated the association between experience and the level of empathy.

 You need to specify what experience is. You are reviewing pharmacy students’ empathy, and usually, pharmacy students are not allowed to work until the end of their studies. What experience do you mean?  You need to explain your experience in the method section.

 12. In the results, you have a subheading as Other. It is not a suitable subheading. Revise it.

 Discussion:

 13. In this sentence:

 According to Klein & Hodges, there are no aptitude differences between males and females that could account for gender disparities in empathy levels.37 They contend that practically everyone may develop greater empathy if they are given the right motivation.

 This sentence is non-relevant to assessing gender and empathy. You should remove or substitute is with a reference related to gender.

 14. In the 3 ^rd^ paragraph of the discussion, you are discussing the relationship between study year and empathy. You discussed 4th-year students, but you should add some explanation about your findings about 2nd-year students with lower empathy scores as well.

 Also, among all the studies that assessed school year and empathy score, many had no differences between empathy scores in different years. Do you still think that it is important? Why did you not talk about sample size here? Maybe studies with bigger sample sizes showed no effect or effect. You need to discuss your findings. Otherwise, a narrative review that neglects many non-significant differences is worthless. What if you had 20 studies and only 1 showed school grade is a predictor? Do you neglect those 19 studies?

 I suggest that you should at least add sample size to the cumulative studies that showed no difference versus the ones that showed the difference. In addition, you need to pay attention to the countries. You have many studies from the US, do the results from the US have homogeneity?

 15. In limitation:

 You stated that: We acknowledge that the included studies are heterogeneous, indicating that additional studies are necessary before reaching any firm conclusions.

 Heterogeneity does not always come from the limited number of studies. It can be due to the cultural differences and the pressure that students experience during their studies. You have to discuss all the possible reasons in the discussion so that in the limitation part, your audience can accept this reason from your side.

 16. In this sentence: Thirdly, considering articles written in various languages would have stimulated new ideas about how pharmacy students’ differences affect empathy levels.

 What do you mean by articles written in various languages? Did you include non-English articles? Or you want to indicate different languages in the empathy questionnaire. You should be cautious here because authors have to use a validated version of tools in a second language to make sure the meaning of the tools has not been changed. Checking JSE tools for empathy, you can see that validated questionnaires exist in different languages.

Are the rationale for, and objectives of, the Systematic Review clearly stated?

Partly

Is the statistical analysis and its interpretation appropriate?

No

Are sufficient details of the methods and analysis provided to allow replication by others?

Partly

Are the conclusions drawn adequately supported by the results presented in the review?

Partly

Reviewer Expertise:

I am pharmacist (Pharm.D) with Master of science in Health research methodology and prior experience in measuring empathy among pharmacy students. My article is cited in this manuscript.

I confirm that I have read this submission and believe that I have an appropriate level of expertise to confirm that it is of an acceptable scientific standard, however I have significant reservations, as outlined above.

# Reviewer report: Reviewer response for version 1

**DOI:** 10.5256/f1000research.139482.r199445

**Published:** 2023-08-25

**Authors:** Logan Thomas Murry

**Affiliations:** 1 College of Pharmacy, The University of Iowa, Iowa City, Iowa, USA

Thank you for the opportunity to review the manuscript “Pharmacy students’ empathy and its determinants: a systematic review.” The discussion of determinants is robust and well-detailed. I have concerns related to the objectives and the study eligibility criteria, specifically why other reviews and qualitative studies were excluded. Below are specific comments and suggestions related to components of the manuscript.

**Abstract:**

 For methods, it might be helpful to include some additional methods reflecting the search strategy such as the dates, specific keywords or MESH terms. This would allow readers to know what period articles were include from and how the total number of articles was identified.

 For results, including the total number of articles identified would help to understand the number of articles which met inclusion criteria.

**Background**

 The background generally provides good rationale for the value added by this study.

 “significance of increasing empathy in health professions students.” Is this intended to mean “despite the importance and increased attention to fostering empathy in health profession students.” ?

 “As personal discomfort from burnout, sadness, and diminished quality of life develops among trainees during their training, they are less likely to feel or show empathy. Neumann *et al.* found that deficits in the formal ( *e.g.*, lack of formal empathy training), informal (inadequate mentors and improper learning environments), and factors outside of the medical curriculum ( *e.g.*, abuse of students and high workload) medical curriculum.”  Medical curriculum repeated twice here.

 The objectives of the study are listed as answering two questions, the first being: 1) How is empathy measured? The title and background place most of the emphasis on determinants of empathy. Additionally, inclusion criteria included later potentially limit articles related to empathy measurement in pharmacy. It may be more appropriate to remove this objective as empathy measurement is not a focus of the methods.

**Methods**

 As mentioned above, I have concerns related to the study objectives and in the eligibility criteria for studies. Additionally, what justification exists for the exclusion of review articles and qualitative studies? Qualitative studies are often useful in developing theoretical frameworks, which would help understand antecedents or contributing factors/domains to empathy development.

 The article assessment and data extraction process are seemingly robust and well-described.

**Results**

 The description of determinants related to pharmacy student empathy was very well done and thorough.

 The section “Outcome Measures” appears to address the first object, How is empathy measured? Importantly, the review identified all articles used self-reported measures. This is likely a result of the inclusion criteria, as other studies have assessed empathy without quantitative self-report measures.

**Discussion**

 “Additionally, survey findings show that encounters with elderly patients at a single event improved students' comfort levels during screening, counseling, and communication.” This statement appears to be missing a citation.

 “Second, because most of the studies that were included measured empathy using self-reports, they did not necessarily accurately capture the empathy of pharmacy students and how patients felt. Understanding how the patient feels and the patient's situation is critical for pharmacy students to grasp the significance of empathy for patients. It is highly advised to conduct qualitative research to obtain a more accurate understanding of the patient's feelings and empathy.”

 Qualitative research on patient feelings and empathy could be included in this review, as there are published qualitative studies looking at empathy. The inclusion of predominately self-reported assessments are potentially a result of the eligibility criteria.

**Conclusions**

 Conclusions are well-written and are appropriate for the study.

Are the rationale for, and objectives of, the Systematic Review clearly stated?

Yes

Is the statistical analysis and its interpretation appropriate?

Not applicable

Are sufficient details of the methods and analysis provided to allow replication by others?

Yes

Are the conclusions drawn adequately supported by the results presented in the review?

Yes

Reviewer Expertise:

Pharmacy Student Development, Qualitative Methods, Empathy, Community Pharmacy Service Evaluation, Pharmacy Education, Continuing Professional Development, ACPE Standard 4 Skill and Key Element Acquisition

I confirm that I have read this submission and believe that I have an appropriate level of expertise to confirm that it is of an acceptable scientific standard, however I have significant reservations, as outlined above.

# Reviewer report: Reviewer response for version 1

**DOI:** 10.5256/f1000research.139482.r162817

**Published:** 2023-03-03

**Authors:** Ahsan Saleem

**Affiliations:** 1 Bolton Clarke Research Institute, Melbourne, VIC, Australia

Thank you for giving me the opportunity to review this manuscript. It’s a systematic review focused on empathy and its determinants in pharmacy students. I have provided my specific comments to improve the quality of the manuscript.

- I have spotted several grammatical and linguistic errors throughout the manuscript; therefore it needs a copy edit.
- Empathy is not something that can be possessed. It can be developed or enhanced, but not possessed. Please rephrase “ *Empathy for others must be possessed by healthcare professionals and healthcare students as healthcare professionals’ candidates, including the pharmacy student*” to “Healthcare professionals and students must have or develop empathy for others.”
- Please remove ‘they’ from “ *but they also result in favorable patient outcomes such as better patient self-care, higher patient satisfaction and faster recovery times.*”
- Please rephrase this sentence “ *We used peer-reviewed original papers, full text, must assess determinants that are associated with pharmacy students' empathy, and only be focused on pharmacy students (first to the fourth year) as healthcare professionals candidates.*”
- The aims mentioned in the abstract are not aligned with the main text of the manuscript.
- Database search was only limited to PubMed, Science Direct, and Scopus. I am wondering why International Pharmaceutical Abstracts was not searched given that the focus of the review is on pharmacy professionals and students.
- Why search duration was limited from July 14, 2022, to July 16, 2022. Is that the date when a search was conducted? What was the actual search duration?
- What is publication age? Are you referring to the date of publication? 
- The findings of this systematic review have been reported under several themes, but I could not find how the authors did the thematic analysis. What was the method used to generate themes?

Are the rationale for, and objectives of, the Systematic Review clearly stated?

Yes

Is the statistical analysis and its interpretation appropriate?

Partly

Are sufficient details of the methods and analysis provided to allow replication by others?

Partly

Are the conclusions drawn adequately supported by the results presented in the review?

Yes

Reviewer Expertise:

Pharmacy practice

I confirm that I have read this submission and believe that I have an appropriate level of expertise to state that I do not consider it to be of an acceptable scientific standard, for reasons outlined above.
